# Health Risk Assessment and Source Apportionment of Mercury, Lead, Cadmium, Selenium, and Manganese in Japanese Women: An Adjunct Study to the Japan Environment and Children’s Study

**DOI:** 10.3390/ijerph17072231

**Published:** 2020-03-26

**Authors:** Chaochen Ma, Miyuki Iwai-Shimada, Nozomi Tatsuta, Kunihiko Nakai, Tomohiko Isobe, Mai Takagi, Yukiko Nishihama, Shoji F. Nakayama

**Affiliations:** 1 Center for Health and Environmental Risk Research, National Institute for Environmental Studies, 16-2 Onogawa, Tsukuba, Ibaraki 305-8506, Japaniwai.miyuki@nies.go.jp (M.I.-S.); isobe.tomohiko@nies.go.jp (T.I.); takagi.mai@nies.go.jp (M.T.); nishihama.yukiko@nies.go.jp (Y.N.); 2 Department of Development and Environmental Medicine, Tohoku University Graduate School of Medicine, Sendai, Miyagi 980-8575, Japan; nakaik@med.tohoku.ac.jp (K.N.); nozomi@med.tohoku.ac.jp (N.T.)

**Keywords:** mercury, cadmium, lead, manganese, selenium, exposure assessment, health risk

## Abstract

Toxic element pollution is a serious global health concern that has been attracting considerable research. In this study, we elucidated the major routes of exposure to three toxic elements (mercury, cadmium, and lead) and two essential elements (manganese and selenium) through diet, soil, house dust, and indoor air and assessed the potential health risks from these elements on women from the coastal area of Miyagi prefecture, Japan. Twenty-four-hour duplicate diet, house dust, soil, and indoor air samples were collected from 37 participants. Cd, Pb, Mn, and Se concentrations were measured using inductively coupled plasma mass spectrometry, and Hg concentrations using cold vapor atomic absorption spectrometry. We found that soil and house dust were the primary reservoirs of these elements. Diet contributed most strongly to the daily intake of these elements, with mean values of 0.72, 0.25, 0.054, 47, and 0.94 μg/kg/day for Hg, Cd, Pb, Mn, and Se, respectively. The mean hazard quotient of Hg was 1.53, indicating a high potential health risk from Hg exposure in daily lives. The intakes of other elements were below the tolerable limits. Future studies with a larger sample size are warranted to confirm our findings.

## 1. Introduction

Toxic metals are naturally occurring ubiquitous substances in the environment that can be released through natural means (e.g., atmospheric deposition and weathering of parent materials) and primarily through anthropological activities (e.g., wastewater irrigation, vehicular exhaust, and solid waste disposal) [1,2]. Typically, metals are categorized into essential and non-essential metals on the basis of their metabolic roles. Essential metals are the necessary nutrients that participate in crucial physiological and biological functions at extremely low concentrations. For example, manganese (Mn) is required for normal brain functioning, bone health, and several enzymatic functions [3], and selenium (Se) supplementation has beneficial effects on the risk of lung and prostate cancer [4,5]. However, supplementation confers benefits only if the nutrient intake is inadequate. For example, excess Mn consumption could cause neurodegenerative diseases [6], and Se supplementation in those with adequate Se intake could increase the risk of type 2 diabetes [7]. 

Because of their low degradability and bioaccumulation, toxic metals are likely to cause chronic toxicity. For example, elevated levels of mercury (Hg) are related to neurological and renal damage [8]; particularly, increased methylmercury concentrations in cord blood have been associated with neurobehavioral and neurodevelopmental deficits in children, due to contamination of fish with methylmercury [9,10,11]. Excess cadmium (Cd) intake can cause kidney disease and hypertension [12,13], and excess lead (Pb) intake is linked to sterility, abortion, stillbirth, and neonatal morbidity, mortality and neurodevelopmental deficits via in utero and/or current exposure [14]. Considering the significant health risks from toxic metals, some international bodies, such as the US Environmental Protection Agency (USEPA), Joint FAO/WHO Expert Committee on Food Additives (JECFA), and the European Commission, have developed guidelines on the intake of toxic metals by humans [15,16]. In Japan, the Food Safety Commission of Japan is responsible for establishing the tolerable intake levels for metals/metalloids (https://www.fsc.go.jp/). In recent years, the tolerable levels of metals/metalloids for the Japanese people have been amended for many times. However, to our knowledge, data on health risks from exposure to metals/metalloids in Japan are still very limited. Among the existing studies in Japan, it has been known that fish and rice are the leading sources of Hg and Cd, respectively [17,18]; food is the primary source of Mn and Se [19,20]. In comparison, the major source of Pb has not been identified [21]. 

Toxic metal-associated health risks have been widely explored, but some of these studies are not without flaws. First, most of them have focused on a single toxic metal or a single exposure route. However, toxic metals can enter the human body via multiple routes, such as oral (such as food, water, soil, and dust), inhalation, and dermal routes [22]. Neglecting to consider various exposure routes could lead to underestimation of the health risks from toxic metals. Second, most studies have assessed the health risks of toxic metals based on total diet studies [22,23], which may not be a sufficient exposure assessment. Third, few studies have examined the source and route of exposure to Cd and Pb especially in pregnant women. Therefore, in this study, we assessed the a) concentrations of three toxic metals (i.e., Hg, Pb, and Cd) and two essential elements (i.e., Mn and Se) in diet, soil, house dust, and indoor air; b) major routes of exposure to these elements; and c) potential health risks from these elements. Additionally, the intake level of toxic elements is commonly low, and few studies focused on the at-risk population. The present study enrolled participants with high blood concentrations of heavy metals. 

## 2. Materials and Methods 

### 2.1. Study Participants 

**Figure 1:**
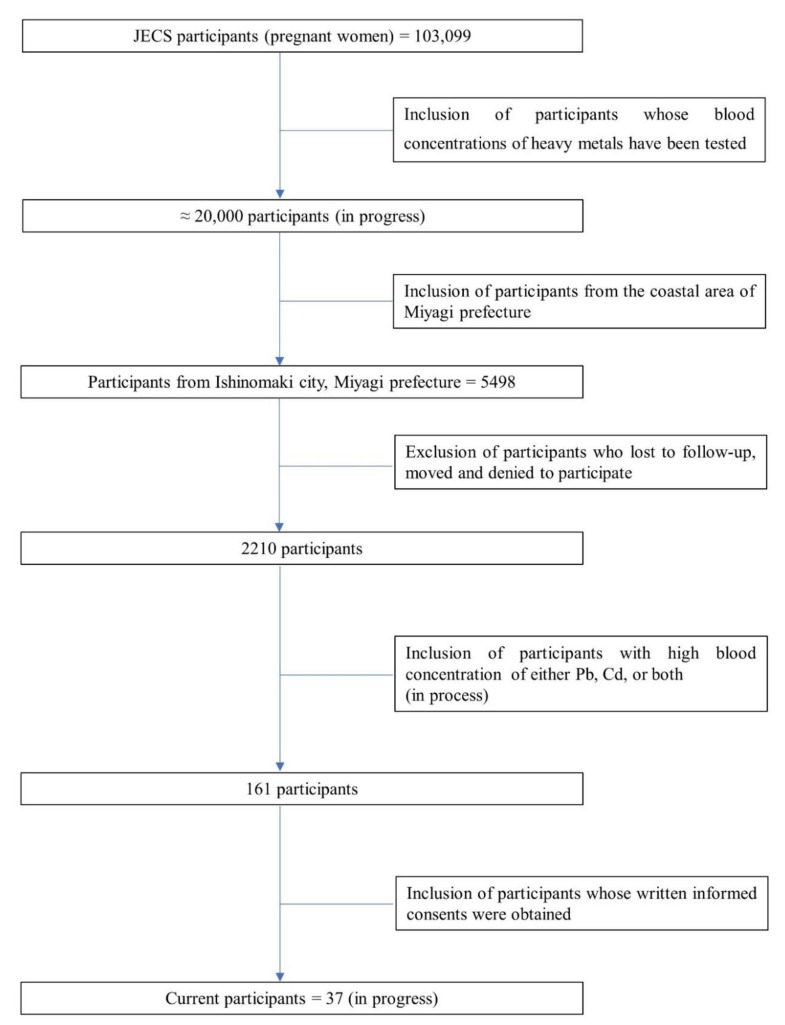
Process of enrollment to this study. The participants were recruited from the coastal area of Miyagi prefecture, Japan.

This survey was conducted as an adjunct study of the Japan Environment and Children’s Study (JECS). The JECS design is described elsewhere [21,24,25]. Briefly, JECS recruited 103,099 pregnant women between January 2011 and March 2014 from 15 study regions covering a wide geographical area in Japan, with a planned follow-up of 13 years. In December 2017, we began registering the current study participants. At that time, JECS had already measured the concentrations of heavy metals in the peripheral blood of 20,000 mothers during mid/late-term pregnancy in 2011–2013. The participants with relatively elevated heavy metal concentrations were recruited to this study. We excluded mothers who moved after delivery because the source and route of exposure might have changed from those during pregnancy. We also excluded mothers who were lost to follow-up or who refused to participate. In this first analysis, we included women with high blood concentrations of Pb and/or Cd from the coastal area of Miyagi prefecture, Japan, as this is a high-risk group. The thresholds for high levels of blood concentrations of Pb and Cd were set at 19.5 ng/g (1.96 μg/dL) and 2.47 ng/g (2.48 μg/L), respectively, which corresponded to the 99.2th percentile of the blood concentration level among the JECS participants. However, if subjects above the set threshold did not provide consent, those with the next highest concentrations were asked to participate. In total, of the 41 women who were interviewed, 37 participated in the study. Figure 1 shows the flowchart of the process of study subject recruitment. The study was approved by Ethical Committee in Institutional Review Boards of National Institute for Environmental Studies (2019-009) (11 October 2018) and Tohoku University Graduate School of Medicine (2018-1-521) (7 November 2019). A written informed consent was obtained from all individual participants included in the study. The characteristics of study subjects are presented in Table S1. 

### 2.2. Sample Collection and Preparation

#### 2.2.1. Diet 

Twenty-four-hour duplicate diet samples, including breakfast, lunch, dinner, snacks, supplements (excluding prescription drugs), tap/filtered water, and other beverages, were collected from each participant on 3 days. Before the investigation, each participant was asked to provide a list containing her frequently consumed food items and then to choose the diet from the list during the sampling days. The duplicate diet samples collected from each participant in polypropylene bags were weighed individually and sent to the laboratory for testing. 

#### 2.2.2. House Dust 

House dust samples (>1 g) were collected using Dyson DC 34 vacuum cleaner (Dyson Ltd., Malmesbury, UK) with a collection bag attached to the tip of the cleaner nozzle. The vacuum dust was passed through 250 μm stainless steel mesh using a mechanical shaker (AS-200, Retsch, Haan, Germany), and the filtered fraction was used for analysis. 

#### 2.2.3. Soil

Soil samples were collected from five sites in the vicinity of each participant’s house by using a pre-cleaned stainless-steel scoop. The sample from each site was kept in a separate polyethylene bag. After air-drying, each sample was passed through a 2 mm mesh sieve, and the filtered fraction was weighed and thoroughly mixed with samples from other sites at the same weight. 

#### 2.2.4. Indoor Air

Indoor air samples were collected using two mini-pumps (MP-Σ300NII, Sibata Scientific Technology Ltd., Tokyo, Japan) with a flow rate of 1.5 L/min operated on a cycle of 5 min on and 30 min off for 1 week (each pump collected 2160 L of air). The particulate matter in the air samples was collected using two sheets of polytetrafluoroethylene (PTFE) filter paper (PF020, ADVANTEC Group, Tokyo, Japan). The weight of the filtrate was measured before and after sampling, and the difference was defined as the amount of particle matter.

### 2.3. Analytical Methods 

To analyze Cd, Pb, Mn, and Se concentrations, an aliquot of each sample (diet, 1 g; soil, 0.1–0.2 g; house dust, 0.5–1 g; and indoor air, two sheets of filter paper) was digested with 3 mL of hydrofluoric acid (HF) and 5 mL of Nitric acid (HNO_3_) using a microwave digestion system. After digestion, HF was removed using 1 mL of hydrogen peroxide (H_2_O_2_) in a Teflon beaker on a hot plate. Then, an internal standard solution was added, followed by addition of ultrapure water up to a certain volume (diet, 50 mL; house dust, 50 mL; soil, 100 mL; and indoor air, 10 mL). Cd, Pb, Mn, and Se concentrations in the digested samples were determined using inductively coupled plasma mass spectrometry (ICP-MS, Agilent 7500cx, Agilent Technologies, Tokyo, Japan) after appropriate dilution for calibration and internal standardization. Hg analysis was conducted using the Hg analysis method of the Ministry of the Environment. For the analysis of Hg concentrations, an aliquot of each sample (diet, 0.2 g; house dust, 10–50 g; and soil, 0.2 g) was mixed with 1 mL of pure water. Then, 2 mL of HClO_4_ and 5 mL of H_2_SO_4_ were added, and the mixture was decomposed by heating at 200–300 °C for 30 min. After cooling, ultrapure water was added to a volume of 50 mL. Hg levels were measured using cold vapor atomic absorption spectrometry (CVAAS, HG-201, Sanso Seisakusho Co. Ltd., Tokyo, Japan). All element analyses were conducted by IDEA Consultants, Inc. (Tokyo, Japan). Table S2 shows the instrumental conditions of ICP-MS and CVAAS. 

### 2.4. Quality Assurance and Quality Control

A vigorous quality control procedure was implemented throughout the analysis by IDEA Consultants. Certified reference materials, i.e., National Institute of Standards and Technology (USA) SRM 2702 (Inorganics in marine sediments) for soil, National Institute for Environmental Studies (Japan) No. 28 (urban aerosol) for indoor air and house dust, and National Research Council Canada (NRC) DORM-4 fish protein for diet, were used. The measured values were in accordance with the certified values (Table S3). The detection limit for each element was calculated using a previously described method [26]. In general, the indoor air concentrations of most elements were lower than the respective limits of detection (LODs). Other measurements were above the LODs. When the concentration was less than the LOD, one-half of the LOD was used for the calculation (Table S4). 

### 2.5. Estimated Daily Intake 

The estimated daily intake (EDI) of each metal through four different routes was calculated using the following equation:

$$\mathrm{EDI}\;=\frac{C\;\times \;D}{\mathrm{BW}}{\mathrm{EDI}}_{M}={\mathrm{EDI}}_{\mathrm{Diet}}+{\;\mathrm{EDI}}_{\mathrm{Soil}}+{\;\mathrm{EDI}}_{\mathrm{House}\;\mathrm{dust}}+{\;\mathrm{EDI}}_{\mathrm{Indoor}\;\mathrm{air}}$$

where C represents the concentration of each element in diet, soil, house dust, and indoor air; D represents the daily consumption through each route; and BW is the body weight of each subject. US EPA’s exposure factors for adults were used to estimate the daily intake rates, namely 16 m^3^/day for air inhalation, 30 mg/day for dust ingestion, and 20 mg/day for soil ingestion [15]. After the respective EDI from each exposure route was calculated, total EDI of each element (EDI_M_) was obtained by summing all of four EDIs.

### 2.6. Risk Assessment 

The hazard quotient (HQ), a ratio of the estimated exposure dose to reference dose, expresses the non-carcinogenic risk of exposure to toxic metals [27]:

$$\mathrm{HQ}=\frac{\mathrm{EDI}}{\mathrm{RfD}}$$

where RfD is the reference dose of daily intake (μg/kg/day), which was 0.1, 1, 4, 140, and 5 μg/kg/day for Hg, Cd, Pb, Mn, and Se, respectively [28]. Furthermore, the hazard index (HI), the sum of the HQs for multiple toxic metals, was applied to evaluate the overall non-carcinogenic risks from five toxic metals [22]:

$${\mathrm{HQ}}_{M}={\mathrm{HQ}}_{\mathrm{Diet}}+{\;\mathrm{HQ}}_{\mathrm{Soil}}+{\;\mathrm{HQ}}_{\mathrm{House}\;\mathrm{dust}}+{\;\mathrm{HQ}}_{\mathrm{Indoor}\;\mathrm{air}}\;\mathrm{HI}={\mathrm{HQ}}_{\mathrm{Hg}}+{\;\mathrm{HQ}}_{\mathrm{Cd}}+{\;\mathrm{HQ}}_{\mathrm{Pb}}+{\;\mathrm{HQ}}_{\mathrm{Mn}}+{\;\mathrm{HQ}}_{\mathrm{Se}}$$

where HQ_M_ is the sum of HQs for a specific element from all exposure routes. HQ or HI > 1 suggests a potential non-carcinogenic risk, whereas HQ or HI < 1 indicates no potential risk. 

## 3. Results 

### 3.1. Concentrations of Five Elements in Diet, Soil, House Dust, and Indoor Air 

The concentrations of each element in diet, soil, dust and indoor air are shown in Table 1, Table 2, Table 3, Table 4, Table 5. The mean concentration of Hg in various sample types decreased in the following order: dust (0.076 μg/g) > soil (0.027 μg/g) > diet (0.0024 μg/g) > indoor air (<LOD). The mean concentration of Cd in various sample types decreased in the following order: dust (1.3 μg/g) > soil (0.34 μg/g) > diet (0.0087 μg/g) > indoor air (<LOD). The mean concentration of Pb in various sample types decreased in the following order: soil (39 μg/g) > dust (28 μg/g) > indoor air (0.022 μg/g) > diet (0.0019 μg/g). The mean concentration of Mn in various sample types decreased in the following order: soil (764 μg/g) > dust (151 μg/g) > diet (1.5 μg/g) > indoor air (<LOD). The mean concentration of Se in various sample types decreased in the following order: dust (0.35 μg/g) > soil (0.23 μg/g) > diet (0.033 μg/g). In general, soil and dust were the primary reservoirs of these elements. 

### 3.2. Daily Intake of Metals and the HQ

The EDIs of the five elements via diet, soil, house dust, and indoor air are presented in Table 6. Diet was the main source of EDI for these elements, with mean values of 0.072, 0.25, 0.054, 47, and 0.94 μg/kg/day for Hg, Cd, Pb, Mn, and Se, respectively. By contrast, indoor air was the least important source of EDI, with mean values of 7.88 × 10^−6^, 8.052 × 10^−5^, 6.5 × 10^−3^, 1.1 × 10^−3^, and 7.06 × 10^−5^ μg/kg/day for Hg, Cd, Pb, Mn and Se, respectively. The highest total EDI was observed for Mn, followed by Se, Cd, Pb, and Hg. The average HQs of Hg, Cd, Pb, Mn, and Se through various exposure routes accounted for 31.2%, 21.9%, 1.9%, 29%, and 16% of HI, respectively, and the mean HI was 1.53 (Table 7). 

## 4. Discussion

### 4.1. Hg

The mean concentration of Hg in soil was 0.027 μg/g, two orders of magnitude lower than that reported in a Chinese study [1]. The mean concentration of Hg in house dust was 0.076 μg/g, one order of magnitude lower than that previously reported in three regions in China [29]. The average EDI of Hg through multiple routes in our study participants was 0.073 μg/kg/day, which is lower than the safety level identified by the USEPA (0.1 μg/kg/day) and Japanese standards (2.0 μg/kg/week or 0.29 μg/kg/day) for pregnant and potentially pregnant women [30]. However, seven (18.9%) participants exceeded the RfD of the USEPA guidelines, and two (5.4%) participants exceeded the Japanese safety levels. Compared with previous dietary studies, the EDI of Hg from diet in our study was lower than that reported in studies conducted in China (0.09 μg/kg/day) [31], but higher than those reported in Chile (0.07 μg/kg/day) [32] and Korea [33]. A possible reason could be that those studies did not consider as many exposure routes. 

**Figure 2:**
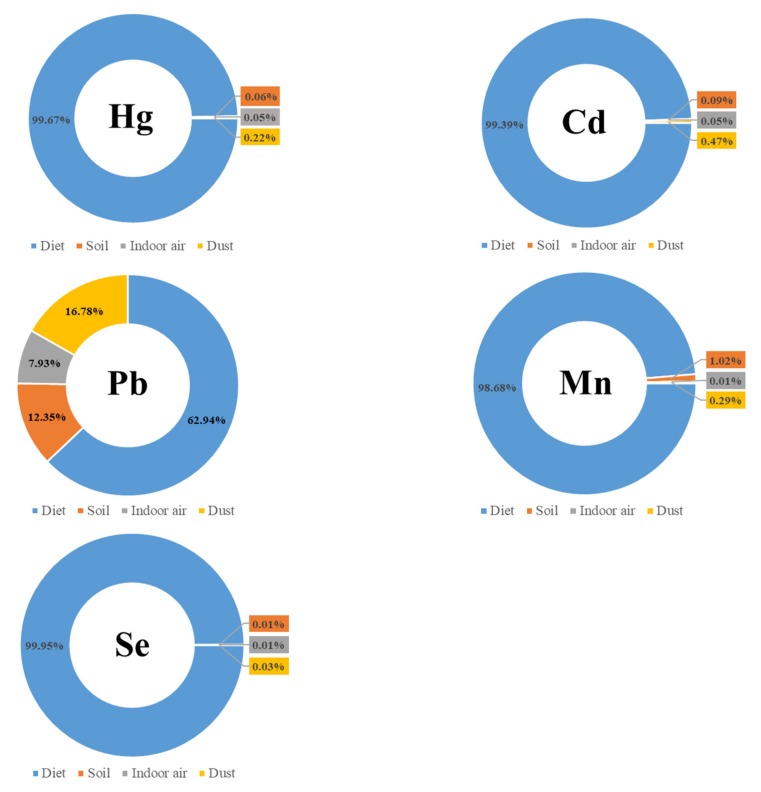
Contribution of each exposure route to Hg, Cd, Pb, Mn, and Se intake.

The average HQ of Hg was 1.53, indicating that this element carries a high non-carcinogenic risk of exposure. Among the multiple exposure routes, diet contributed most strongly to the daily intake of Hg (99.7%), followed by house dust (0.22%), soil (0.062%) and air (0.048%) (Fig. 2). The dominant role of diet in Hg intake is consistent with the results of a Chinese study [34]. 

### 4.2. Cd

The mean concentration of Cd in soil was 0.34 μg/g, which was lower than some regions in China [1,34,35]. The mean concentration of Cd in house dust was 1.3 μg/g, similar to that reported in a previous Japanese study [36] and 40-fold lower than that recorded in a region in China [22], whereas the value was higher than that reported in a study in Turkey [37]. The average total EDI of Cd through multiple routes was 0.25 μg/kg/day, which was much lower than the tolerable intake level identified by the USEPA (1 μg/kg/day) [15] and the Japanese standard (1 μg/kg/day) [30]. The EDI of Cd via diet in this study was lower than those reported in previous duplicated diet studies in Japan (2.08 μg/kg/week) [38] and Portugal (0.73 μg/kg/day) [39], but slightly higher than that recorded in a Chinese study (1.49 μg/kg/week) [40]. 

The average HQ of Cd was 0.25, and the maximal value was also <1 (0.86), indicating the lack of a non-carcinogenic risk of exposure to Cd. This value was lower than previously reported findings [22,34,35]. Among the exposure routes, diet contributed most strongly to daily Cd intake (99.4%), followed by dust (0.47%), soil (0.087%), and indoor air (0.053%) (Figure 2). This finding is basically consistent with previous studies [22,41], but is different from that of another study [34], in which air was the main Cd intake source.

### 4.3. Pb

The median Pb concentration in soil was 19 μg/g, exceeding that reported in a previous Japanese study (12.2 μg/g) [42], but much lower than that obtained in a previous Japanese investigation [43], and some studies in China [1,34,35]. The mean Pb concentration in house dust was 28 μg/g, which was considerably lower than those reported in studies from Japan (94.5 μg/g) [43], the United Kingdom (181 μg/g) [44], Australia (389 μg/g) [45], China (1467 μg/g) [22], and Vietnam (549 μg/g) [41]. The mean concentration of Pb in indoor air was 0.023 μg/m3, which was remarkably lower than that in a Chinese study [34]. The average total EDI of Pb through multiple routes was 0.09 μg/kg/day, noticeably below the tolerable intake level of 4 μg/kg/day [28], and there is no reference value in the Japanese standard. The EDI of Pb via diet in this study was lower than those reported in Chile (3.3 μg/kg/day) [32], Korea (0.41 μg/kg/day) [33], China (1.26 μg/kg/day) [40], France (0.2 μg/kg/day) [46], and Spain [47]. 

The average HQ of Pb ranged from 0.0084 to 0.092, revealing the absence of non-carcinogenic risk of exposure to this element. Moreover, our average HQ of Pb was considerably lower than those reported previously [1,22,34]. Among the exposure routes, diet mostly strongly contributed to the daily intake of Pb (62.9%), followed by house dust (16.8%), soil (12.3%), and indoor air (8%) (Figure 2). This finding is inconsistent with those of previous studies, in which either soil or air was the main intake source [34,41]. The difference may have resulted from the unique characteristics of the study areas (e.g., e-waste or mining area). Thus, more research is needed to elucidate these issues. 

### 4.4. Mn

The mean Mn concentration in house dust was 151 μg/g, slightly higher than that in a study from Turkey (136 μg/g) [37], but lower than that in a study from Canada (267 μg/g) [48]. The average dietary intake of Mn in this study was 2567 μg/day, which was lower than the values reported in a previous Japanese study (4900 μg/day) [19], and a Polish study (4460 μg/day [22], but slightly higher than that recorded in a study performed in Italy (2340 μg/day). The total intake of Mn was 2587 μg/day, which was lower than the tolerable daily intake in Japan (11,000 μg/day) [49], whereas it was higher than the appropriate intake recommended for women (1800 μg/day) [50]. The average EDI of Mn through multiple routes was 47 μg/kg/day, which was considerably lower than the Japanese standard (180 μg/kg/day) [42], and USEPA limit (140 μg/kg/day) [51].

The HQ of Mn ranged from 0.067 to 0.86, indicating the absence of non-carcinogenic risk associated with exposure to Mn. Among the exposure routes, diet contributed most noticeably to the daily intake of Mn (98.7%), followed by soil (1%), house dust (0.29%), and indoor air (0.003%) (Figure 2). This finding is consistent with results from a Vietnam study [41]. More studies are warranted to evaluate this issue.

### 4.5. Se

The average dietary intake of Se of this study was 51.9 μg/day, two-fold the recommended daily intake in Japan (25 μg/day) [49]. Our result was lower than the findings from a study from Italy (66.53 μg/day) [52], but slightly higher than results from a Poland (46.8 μg/day) study [23]. The average EDI of Se through multiple routes was 0.94 μg/kg/day, which was much lower than the Japan (4 μg/kg/day) [49] and USEPA-recommended daily intake limit (5 μg/kg/day). 

The HQ of Se ranged from 0.027 to 0.34, indicating that lack of non-carcinogenic risk of exposure to Se. Among the exposure routes, diet contributed most greatly to the daily intake of Se (>99.9%), and the remaining exposure routes accounted for <0.01% (Figure 2).

In general, the total EDIs of these metals did not exceed the safety thresholds. However, the maximum total EDI of Hg exceeded the RfD (Table 7). The average HI of these five metals was 1.5, indicating there is a potential risk associated with exposure to these five elements. 

Some limitations of this study should be acknowledged. Firstly, the sample size is small. However, the participants of this study were from the subsample of JECS, which means the results may provide a general picture of the daily intakes of these elements for the Japanese pregnant women. Secondly, we only considered four exposure routes for each element. Intake from other sources may have been neglected. Indoor air metal concentration indicated metal concentrations in trapped particle using air sampler. For this reason, mercury vapor was not considered in this study. Thirdly, study participants were not enrolled at the same time, which may have resulted in seasonal variation in dietary intake and introduced uncertainty into the risk assessment. Fourthly, the lack of data on specific food items prevented the identification of foods with the highest elemental concentrations. Fifthly, study participants were only from Miyagi prefecture, future studies are needed to explore whether there is geographical difference in health risks due to these elements. 

## 5. Conclusions

This was an adjunct study of JECS focusing on women with high blood concentrations of Cd and/or Pb. We evaluated the health risks associated with exposure to Hg, Pb, Cd, Mn, and Se via multiple routes in daily life. Furthermore, we elucidated the main source of intake for each element. On average, we found low health risks associated with exposure to Cd, Pb, Mn, and Se but a high potential risk from exposure to Hg. Among the multiple exposure routes, diet contributed most strongly to the intake of these elements. However, the estimated risk was generally lower than that reported in other countries.

## Figures and Tables

**Table 1:** Summary of Hg concentrations according to the exposure route.

Route (N = 37)	Range	P5	P25	Median	P75	P95	Mean	SD
Diet (μg/g wet)	0.00022–0.018	0.00024	0.0004	0.00094	0.0017	0.011	0.0024	0.004
Soil (μg/g dry)	0.0032–0.072	0.0093	0.018	0.024	0.031	0.067	0.027	0.017
House dust (μg/g)	0.022–0.25	0.026	0.045	0.065	0.096	0.15	0.076	0.048
Indoor air (μg/m^3^)	LOD–0.000087	LOD	LOD	LOD	LOD	LOD	LOD	LOD

LOD: limit of detection; SD: standard deviation.

**Table 2:** Summary on Cd concentrations according to the exposure route.

Route (N = 37)	Range	P5	P25	Median	P75	P95	Mean	SD
Diet (μg/g wet)	0.0011–0.036	0.0036	0.0051	0.0069	0.011	0.015	0.0087	0.0060
Soil (μg/g dry)	0.13–1	0.16	0.23	0.28	0.4	0.64	0.34	0.18
House dust (μg/g)	0.03–29	0.12	0.26	0.45	0.68	1.42	1.3	4.7
Indoor air (μg/m^3^)	LOD–0.0015	LOD	LOD	LOD	LOD	0.00052	LOD	LOD

LOD: limit of detection; SD: standard deviation.

**Table 3:** Summary on Pb concentrations according to the exposure route.

Route (N = 37)	Range	P5	P25	Median	P75	P95	Mean	SD
Diet (μg/g wet)	0.00074–0.0046	0.00085	0.0011	0.0016	0.0022	0.0038	0.0019	0.00095
Soil (μg/g dry)	9.7–740	12	16	19	23	33	39	118
House dust (μg/g)	2.4–94	6.7	10	17	34	78	28	25
Indoor air (μg/m^3^)	LOD–0.11	LOD	0.0052	0.014	0.029	0.064	0.022	0.023

LOD: limit of detection; SD: standard deviation.

**Table 4:** Summary on Mn concentrations according to the exposure route.

Route (N = 37)	Range	P5	P25	Median	P75	P95	Mean	SD
Diet (μg/g wet)	0.45–2.4	1.07	1.2	1.6	1.8	2.2	1.5	0.41
Soil (μg/g dry)	360–3900	422	590	690	830	968	764	555
House dust (μg/g)	5–640	38	87	130	200	278	151	111
Indoor air (μg/m^3^)	LOD–0.01	LOD	LOD	LOD	LOD	0.007	LOD	LOD

LOD: limit of detection; SD: standard deviation.

**Table 5:** Summary on Se concentrations according to the exposure route.

Route (N = 37)	Range	P5	P25	Median	P75	P95	Mean	SD
Diet (μg/g wet)	0.015–0.066	0.017	0.024	0.031	0.039	0.054	0.033	0.012
Soil (μg/g dry)	0.071–0.53	0.13	0.16	0.21	0.28	0.4	0.23	0.091
House dust (μg/g)	0.11–1.7	0.16	0.24	0.28	0.39	0.54	0.35	0.26
Indoor air (μg/m^3^)	LOD–0.0031	LOD	LOD	LOD	0.00026	0.00041	0.00026	0.00049

LOD: limit of detection; SD: standard deviation.

**Table 6:** Estimated daily intake of five elements.

Element (N = 37)	Diet (μg/kg/day) Mean ± SD Range	Dust (μg/kg/day) Mean ± SD Range	Soil (μg/kg/day) Mean ± SD Range	Air (μg/kg/day) Mean ± SD Range	Total EDI ^a^ (μg/kg/day) Mean ± SD Range
Hg	7.2 × 10^−2^ ± 1.4 × 10^−1^ 2.5 × 10^−3^ – 6.5 × 10^−1^	4.17 × 10^−5^ ± 2.67 × 10^−5^ 8.31 × 10^−6^ – 1.5 × 10^−4^	1.02 × 10^−5^ ± 6.35 × 10^−6^ 1.016 × 10^−6^ – 2.72 × 10^−5^	7.89 × 10^−6^ ± 4.01 × 10^−6^ 3.13 × 10^−6^ – 2.8 × 10^−5^	7.3 × 10^−2^ ± 1.4 × 10^−1^ 2.5 × 10^−3^ – 6.5 × 10^−1^
Cd	2.5 × 10^−1^ ± 1.6 × 10^−1^ 1.8 × 10^−2^ – 8.6 × 10^−1^	6.4 × 10^−4^ ± 2.3 × 10^−3^ 1.67 × 10^−5^ – 1.4 × 10^−2^	1.3 × 10^−4^ ± 7.34 × 10^−5^ 4.13 × 10^−5^ – 4.3 × 10^−4^	8.052 × 10^−5^ ± 6.47 × 10^−5^ 3.17 × 10^−5^ – 4.2 × 10^−4^	2.5 × 10^−1^ ± 1.6 × 10^−1^ 1.9 × 10^−2^ – 8.6 × 10^−1^
Pb	5.4 × 10^−2^ ± 2.7 × 10^−2^ 1 × 10^−2^ – 1.2 × 10^−1^	1.5 × 10^−2^ ± 1.3 × 10^−2^ 1.3 × 10^−3^ – 5.5 × 10^−2^	1.5 × 10^−2^ ± 4.6 × 10^−2^ 2.8 × 10^−3^ – 2.9 × 10^−1^	6.5 × 10^−3^ ± 6.7 × 10^−3^ 6.5 × 10^−4^ – 2.9 × 10^−2^	9 × 1 0^−2^ ± 5.7 × 10^−2^ 3.3 × 10^−2^ – 3.7 × 10^−1^
Mn	47 ± 22 7.45 – 120	8.2 × 10^−2^ ± 6.3 × 10^−2^ 2.8 × 10^−3^ – 3.7 × 10^−1^	2.8 × 10^−1^ ± 2.2 × 10^−1^ 1.4 × 10^−1^ – 1.51	1.1 × 10^−3^ ± 0 3.3 × 10^−4^ – 3.3 × 10^−3^	47 ± 22 9.34 – 120
Se	9.4 × 10^−1^ ± 3.3 × 10^−1^ 1.3 × 10^−1^ – 1.68	1.9 × 10^−4^ ± 1.2 × 10^−4^ 6.43 × 10^−5^ – 7.3 × 10^−4^	8.36 × 10^−5^ ± 3.61 × 10^−5^ 2.25 × 10^−5^ – 2.1 × 10^−4^	7.06 × 10^−5^ ± 1.1 × 10^−4^ 1.88 × 10^−5^ – 7.1 × 10^−4^	9.4 × 10^−1^ ± 3.3 × 10^−1^ 1.3 × 10^−1^ – 1.68

^a^ EDI: estimated daily intake; SD: standard deviation.

**Table 7:** Hazard quotient and hazard index.

Element	RfD ^a^ (μg/kg/day)	Food Safety Commission of Japan (2015) ^b^ (μg/kg/day)	HQ ^c^ Mean ± SD Range	HI ^d^ Mean ± SD Range
Hg	0.1	0.29 ^e^	1.53 ± 1.47 0.26–7.36	1.53 ± 1.47 0.26–7.36
Cd	1	1	0.25 ± 0.16 0.019–0.86	
Pb	4	NR ^f^	0.023 ± 0.014 0.0084–0.092	
Mn	140	180	0.34 ± 0.16 0.067–0.86	
Se	5	4	0.19 ± 0.066 0.027–0.34	

^a^ RfD: reference dose of daily intake (μg/kg/day) [15,27,28,51,53]. ^b^ The Food Safety Commission of Japan, 2015. ^c^ HQ: hazard quotient. ^d^ HI: hazard index. ^e^ Converted from 2.0 μg/kg/week [30]. ^f^ NR: Not reported; SD: standard deviation.

## Supplementary Materials

The following are available online at https://www.mdpi.com/1660-4601/17/7/2231/s1, Table S1: Basic information for participants (N = 37); Table S2: Analytical instruments; Table S3: Analytical results (mg/kg) of certified reference materials; Table S4: Limit of detection (LOD) of metals for each sample type.
