# Supplementary material for: Health Risk Assessment and Source Apportionment of Mercury, Lead, Cadmium, Selenium, and Manganese in Japanese Women: An Adjunct Study to the Japan Environment and Children’s Study
Source: Int J Environ Res Public Health. 2020 Mar 26;17(7):2231. doi: 10.3390/ijerph17072231 (PMC7177228; doi:10.3390/ijerph17072231)
Supplement: Supplementary file 1 [file ijerph-17-02231-s001.pdf]

Supplementary Material

Table 1. Basic information for participants (N = 37).

Personal Information	Median (25th–75th)	Mean (SD)	Range
Daily food consumption (g)	1678.2 (1288.9–2018.8)	1696.5 (657.6)	391–3529.5
Height (cm)	157 (155–161)	157.4 (4.6)	148–168.2
Body weight (kg)	54 (51–59)	55.7 (9.4)	41–85
BMI	22.2 (20.8–23.3)	22.5 (3.5)	16.2–32

Table 2. Analytical instruments.

**Mn, Se, Cd, Pb: ICP-MS（7500 cx, Agilent Technologies）**	
RF power: 1,550 W	
Carrier gas: 0.90 L/min	
Make-up gas: 0.20 L/min	
Spray chamber temperature: 2 °C	
Reaction gas: He, 4.0 L/min (Mn); H_2 4.4 L/min (Se)	
Monitored ion (m/z)	
Mn: 55	Ge (internal): 72
Se: 78	Ge (internal): 72
Cd: 111	Ge (internal): 72
Pb: 208	Tl (internal): 205
**Hg: CVAAS (Automatic Mercury Analyzer HG-201, Sanso)**	
Method: Cold vapor atomic absorption spectrometry	
Wavelength: 253.7 nm	
Flow: 1–1.5 L/min	

ICP-MS: inductively coupled plasma mass spectrometry; CVAAS: cold vapor atomic absorption spectrometry.

Table S3. Analytical results (mg/kg) of certified reference materials.

		Mn	Se	Cd	Pb	Hg
NIST SRM 2702	Measured	1735	5.36	0.823	133.2	-
	Certified (acceptable)	1757 (1699–1785)	4.95 (4.49–5.41)	0.817 (0.806–0.828)	132.8 (131.7–133.9)	-
NIES No. 28	Measured	702	14.4	5.98	424	-
	Certified (acceptable)	686 (644–728)	14.4 (-)	5.6 (5.17–6.03)	403 (371–435)	-
NRC DORM-4	Measured	3.28	3.63	0.310	0.448	0.389
	Certified (acceptable)	3.17 (2.91–3.43)	3.45 (3.05–3.85)	0.299 (0.281–0.317)	0.404 (0.342–0.466)	0.412 (0.376–0.448)

NIST: Naional Institute of Standards and Technology; NIES: National Institute for Environmental Studies; NRC: National Research Council Canada.

Table S4. Limit of detection (LOD) of metals for each sample type.

		Mn		Se		Cd		Pb		Hg	
**Sample**	**Unit**	**LOD**	**≥LOD (%)**	**LOD**	**≥LOD (%)**	**LOD**	**≥LOD (%)**	**LOD**	**≥LOD (%)**	**LOD**	**≥LOD (%)**
Soil	mg/kg dry	0.04	100	0.007	100	0.0006	100	0.06	100	0.0002	100
Food	μg/g wet	0.002	100	0.00006	100	0.00005	100	0.0004	100	0.0002	100
Air	ng/m^3	7	5.4	0.2	37.8	0.4	13.5	5	75.7	0.05	2.7
Dust	mg/kg	0.1	100	0.001	100	0.0002	100	0.003	100	0.001	100

Indicates the LOD when the sampling volume was 1000 m^3 (LOD depends on the pump volume). When the concentration was less than the LOD, one-half of the LOD was used for the calculation. Indicates the LOD when sample amount used for analysis was 0.05 g.
